# Current controlled switching of impedance in magnetic conductor with tilted anisotropy easy axis and its applications

**DOI:** 10.1038/srep36180

**Published:** 2016-10-26

**Authors:** Mihail Ipatov, Valentina Zhukova, Arkady Zhukov, Julian Gonzalez

**Affiliations:** 1 Dpto. de Física de Materiales, Fac. Qímicas, University of the Basque Country UPV/EHU, San Sebastian 20018, Spain; 2 Dpto. Física Aplicada 1, University Polytechnic School, University of the Basque Country UPV/EHU, San Sebastian 20018, Spain; 3 Ikerbasque, Basque Foundation for Science, Bilbao 48011, Spain

## Abstract

We present a concept and prototype of a memory element based on *current driven* magneto-impedance (MI) effect that stores the binary data (0, 1) as the orientation of the magnetization. The magnetization orientation in the surface layer with tilted anisotropy easy axis can be switched controllably between two stable states by applying current pulses of the appropriate sign, and can be detected by sensing the impedance. We demonstrated the functioning of a non-volatile magnetic memory with a read speed performance up to and above 2 GHz. A prototype of a memory element was realized on a short piece of amorphous microwire, as this material exhibits the highest MI effect, and the required anisotropy can be quite easily obtained. Nevertheless, this concept can be extended to other materials and geometries exhibiting MI effect and possessing a required magnetic anisotropy.

Magnetic random access memory (MRAM) is currently being intensively studied from both scientific and technological perspectives as a next-generation non-volatile memory^1^,^2^,^3^,^4^. Most of the currently applied MRAM types are based on the magneto-resistance (MR) effect^1^. Here we propose to apply another physical effect, the magneto-impedance (MI) one. In contrast to the multilayer MR memories, the MI MRAM is much simpler – it can consist just of a single magnetic layer, and is capable to operate at a GHz frequencies.

The MI effect has attracted much attention, primarily because of its application in low-cost and high sensitive magnetic sensors^5^,^6^,^7^,^8^. Particularly, a sensitivity approaching a pico-Tesla level was reported^9^,^10^ that is the highest among all non-cryogenic sensors. One of the main challenges in the MI sensors is the hysteresis reduction that often appears because of anisotropy easy axis deviation from transversal plane^11^. On the other hand, it was recently shown that if a high tilted anisotropy is induced in a wire, the MI dependence can exhibit considerable hysteresis^12^,^13^,^14^,^15^,^16^,^17^, which was proposed to apply in MRAM^18^. The main drawback of that approach was a need of external magnetic field to read out the stored information.

Here we propose another approach of MI MRAM that is schematically shown in Fig. 1. The MI effect is usually treated as a dependence of the conductor impedance *Z* on external magnetic field *H*_*E*_. On the other hand, the magnetic state, and therefore the impedance can be sensitive to the circular bias field *H*_*B*_ created by static current *I*_*B*_ flowing though a wire with tilted surface anisotropy^19^. In this case the impedance dependence *Z*(*I*_*B*_) is a hysteretic function. No generation of external magnetic field is required to perform the write/store/read operations. The writing of the data bit (switching between two stable magnetization states) is performed by passing a pulse of current of positive or negative polarity sufficiently high to produce irreversible switching of the static magnetization. This method of writing data is commonly used in MR memory^1^, and therefore, similar performance characteristics, such as a long (more than 20 years) data retention and unlimited write endurance can be expected. We demonstrate here that the stored information can be non-destructive read out by sensing the impedance of the memory element. As no power is required to store the information, the memory is also a non-volatile. We experimentally realized such a *current-driven* MI memory element on a piece of amorphous microwire with induced tilted anisotropy and demonstrated its functioning in the frequency range from 10 MHz to more than 2 GHz. The demonstrated current controlled impedance switching can also find applications in microwave devices such as tunable filters, resonators, delayed lines, impedance matching lines etc.

## Model

**Figure 1:**
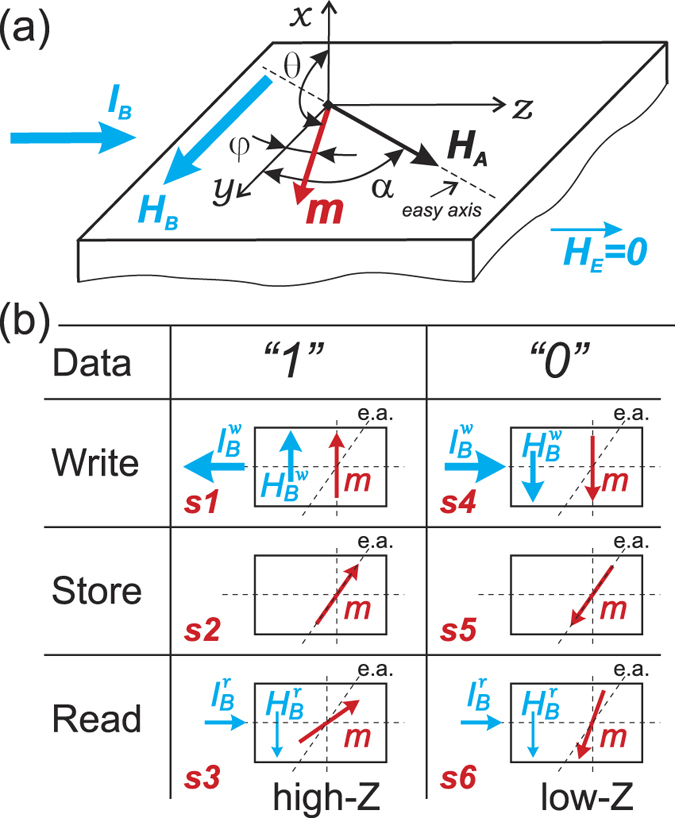
Schematics (**a**) and principle (**b**) of the current controlled impedance switching. The states *s1*–*s6* corresponds to the states of the write/store/read cycle operations.

**Figure 2:**
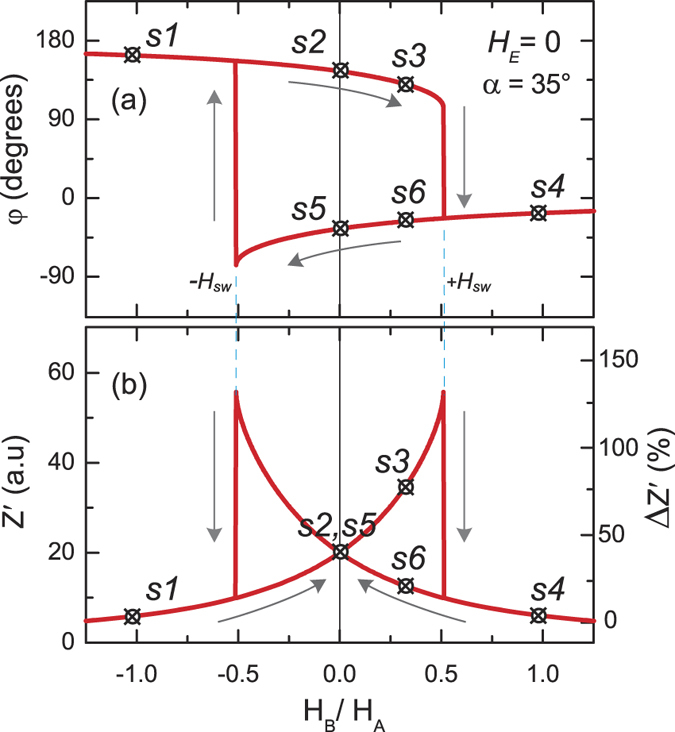
*φ* and *Z*′ dependences on *H*_*B*_ calculated for anisotropy angle *α* = 35°.

The principle of proposed *current-driven* MI memory element is based on (i) the static magnetization switching between two stable states by the static bias current *I*_*B*_ applied to the conductor and (ii) the dependence of high frequency impedance *Z* on the magnetization orientation as schematically shown in Figs 1 and 2.

Let us see a model of magnetization rotation angle *φ* and impedance *Z* dependencies on the static bias field *H*_*B*_ in a wire with a high tilted anisotropy. It was demonstrated that the magnetic structure of the central part of the surface layer in a twisted wire is a mono-domain one^13^. Then, assuming homogenous magnetic structure, the total energy *U* can be expressed as the sum of the magnetostatic energy, the energy of magnetic anisotropy and the energy of applied magnetic field, and an equitation of total energy *U* in the spherical system of coordinates as shown in Fig. 1(a) can be written as^12^:


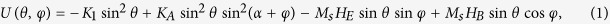


where 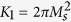, *θ* and *φ* are the polar and azimuthal angle of the magnetization vector, *K*_*A*_ is the anisotropy constant, *H*_*E*_ is the external magnetic field applied along the *z* axis, *H*_*B*_ is the perpendicular (along the *y* axis) static bias field that is produced by the current *I*_*B*_ running through the wire: *H*_*B*_ = *I*_*B*_/(2*πr*) at the wire surface, *r* is wire radius, and *α* is the tilt angle – the deviation angle of the magnetic anisotropy easy axis from transversal direction as shown in Fig. 1(a). The total energy minimum is reached when the magnetization vector lies in plane, i.e. *θ* = *π*/2. Then the Eq. 1 minimizes to:


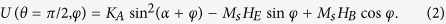


The magnetization orientation can be found by minimizing the total energy *U* over the angle *φ*. The equilibrium angle *φ* between the magnetization vector and the transversal direction is calculated from Eq. 2 as *dU*/*dφ* = 0:


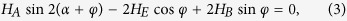


where *H*_*A*_ = 2*K*_*A*_/*M*_*s*_ is the surface anisotropy field.

The applied magnetic field terms *H*_*E*_ and *H*_*B*_ counterbalance the anisotropy field *H*_*A*_ leading to a change of magnetization rotation angle *φ*. As the applied magnetic field, it is usually considered the external axial magnetic field *H*_*E*_. Here, our purpose is to develop a material that does not require any external magnetic field to change its impedance. Therefore we set *H*_*E*_ = 0 and, apply only a circular bias magnetic field *H*_*B*_ created internally in the wire by a bias current *I*_*B*_ to be able to control the magnetic state and thus, the impedance of the wire. Finally, the equation for equilibrium energy can be presented in the following form:


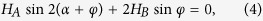


Equation 4, that is a modification of the Stoner-Wohlfarth model^20^, describes the magnetization reversal process under the action of the circular bias field *H*_*B*_. The solution of Eq. 4 in the form *φ*(*H*_*B*_) for a non-zero angle *α* is shown in Fig. 2(a). And, the impedance dependence *Z*(*H*_*B*_) can be calculated using a formula for a strong skin effect^21^:


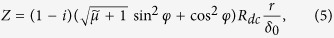


where *R*_*dc*_ is the resistance to direct current, 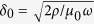 is the non-magnetic skin depths at frequency *ω*, and 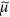 is the relative effective transverse permeability:


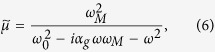


where *ω*_*M*_ = *γμ*_0_*M*_*S*_, *ω*_0_ is ferromagnetic resonance frequency, *α*_*g*_ is the Gilbert damping constant.

Using the obtained above *φ*(*H*_*B*_) dependence and Eqs 5 and 6 we calculated the impedance dependence *Z*(*H*_*B*_) that is shown in Fig. 2(b). In the calculation we used the anisotropy angle *α* = 35° obtained previously for this wire^19^, the other calculation details are given in ref. 19. As one can see, the impedance dependence *Z*(*H*_*B*_) exhibits a hysteresis below the switching field *H*_*sw*_ where the switches from a high impedance to a low impedance are observed.

Further, we describe a principle of the magnetic memory element based on this hysteresis as shown in Figs 1 and 2. Independently of the initial state, after applying a negative current pulse sufficiently high to produce irreversible switching 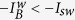 (state *s1*), the magnetization will orient along the easy axis in *Up* direction (state *s2*) which corresponds to the *store logical ‘1’* state. The magnetization states *Up* – *s2* and *Down* – *s5*, both at *I*_*B*_ = 0, are characterized by antiparallel magnetization orientation along the easy axis (see Fig. 2(a)). However, as one can see from the modeled dependence *Z*′(*H*_*B*_) shown in Fig. 2(b), at *H*_*B*_ = 0 the *Z*(*H*_*B*_) branches cross each other. To distinguish between these two equiimpedance states *s2* and *s5*, it is proposed to apply the circular bias field *H*_*B*_ created by static current *I*_*B*_. A small read current 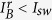 applied to the sample makes the magnetization reversibly rotate. If previously the *Up* state was written, then the read current 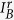 makes the magnetization rotate in the *close-to-axial* direction which exhibits a higher impedance (state *s3*). To set the *Down* state one needs to pass a positive current pulse 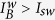 (state *s4*) that, after *I*_*B*_ removal, makes the magnetization orient along the easy axis in *Down* direction (state *s5*) which corresponds to the *store logical ‘0’* state. Now, the application of read current 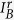 makes the magnetization rotate in the *close-to-transversal* direction which exhibits a lower impedance (state *s6*). In this way, the write/store/read cycle can be realized.

## Results

**Figure 3:**
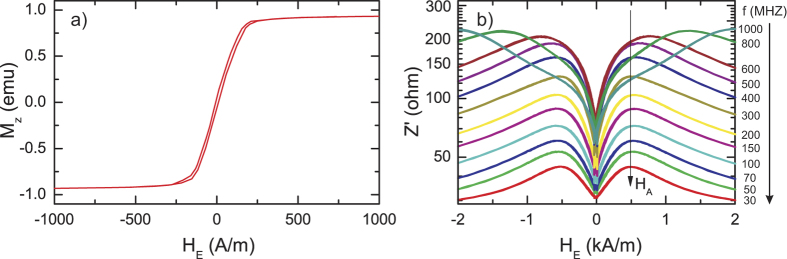
Magnetization *M*_*z*_ (**a**) and impedance *Z*′ (**b**) dependencies as a function on axial external magnetic field *H*_*E*_.

Further we experimentally demonstrate the functioning of the MI memory element. High and sensitive MI effect requires certain well established magnetic anisotropy and small anisotropy constant. For instance, the MI effect has been recently investigated in perovskite magnetic oxides^22^,^23^. The reported values of MI effect are in the range of tens percents in the frequency range from a few thousands kHz to MHz that limits their application to only low access speed ones. Also, a relatively high magnetic anisotropy constant results that a high magnetic field (above tens of kA/m) is required to reach the maximum of MI change that is a disadvantage for memory applications. Therefore, we used amorphous glass-coated microwires^24^ with a small negative magnetostriction that are known to exhibit a very high (more than 500%^5^) MI effect. The wire length was 5 mm, metallic core radius *r* was 10.7 *μ*m, the glass coating thickness was 2.4 *μ*m, and the nominal composition was Co_67.1_Fe_3.8_Ni_1.4_Si_14.5_B_11.5_Mo_1.7_. The wire was twisted and pulled when being soldered to induce the tilted anisotropy in the surface layer of the wire. The hysteresis loop for this wire is shown in Fig. 3(a). One should take into account that the magnetic structure of these wires is a rather complex, it consists of (i) the central core with dominant axial magnetic anisotropy, (ii) the outer shell with a circular or helical anisotropy and (iii) the intermediate layer; and each component contributes differently in the measured hysteresis loop. The volume of outer shell, where the high frequency current is concentrating due to the skin-effect, is relatively small and it can be difficult to determine the surface layer anisotropy from the hysteresis loop of the entire wire. On the other hand, it is known that the MI dependencies *Z*(*H*_*E*_) at intermediate frequencies of 10–500 MHz (bellow the frequencies where the ferro-magnetic resonance (FMR) dominates the MI dependencies) exhibit maximum at the anisotropy field of surface layer^25^. Also, *H*_*A*_ can be extracted from the evolution of the FMR peak field dependence^26^. Thus, from Fig. 3(b) that shows the *Z*′(*H*_*E*_) dependencies for different frequencies from 30 MHz to 1 GHz, the surface anisotropy field *H*_*A*_ was found to be about 500 A/m. As one can see, the observed MI effect Δ*Z*′/*Z* of the *Z*′(*H*_*E*_) dependence is up to 300%. We have observed the MI effect up to 700% in this wire in the unstressed state^14^. This reducing in the MI effect is related with the increasing of magnetic hardness due to induced by twisting additional stress. The reported values of theoretical maximum of MI effect are much higher, up to 3000%^12^,^27^.

**Figure 4:**
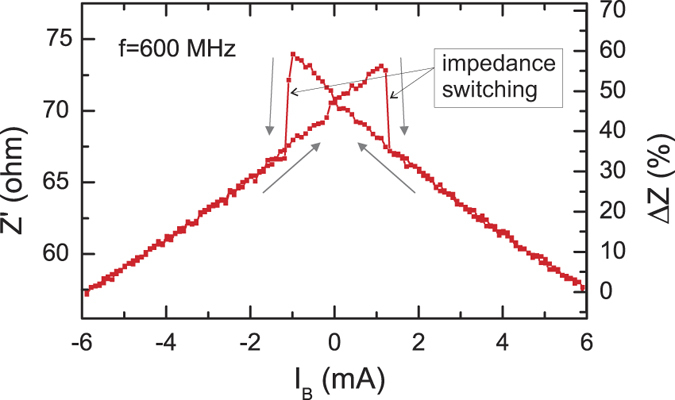
Experimental dependence *Z*′(*I*_*B*_) at *f* = 600 MHz.

Figure 4 shows the measured impedance dependence *Z*′(*H*_*B*_) that qualitatively agrees with the model. The experimentally observed switching current *I*_*sw*_ was about 1.3 mA.

**Figure 5:**
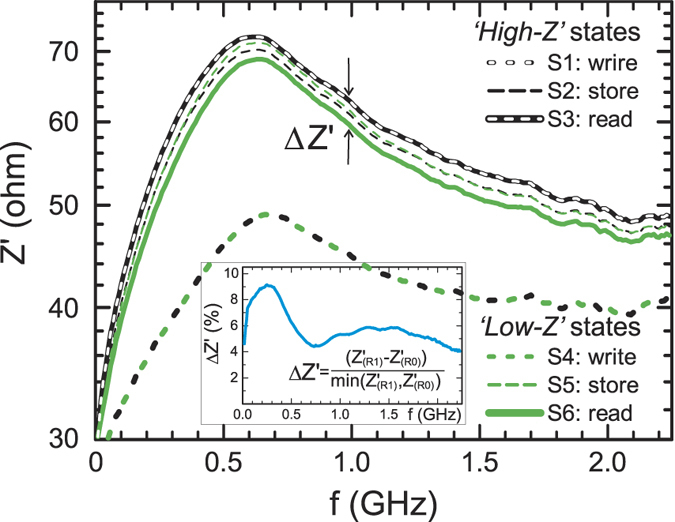
Experimental dependence *Z*′(*f*) with *I*_*B*_ as a parameter. The inset shows the impedance difference Δ*Z*′ between *read 1* and *read 0* curves.

Further we selected a number of current values which corresponds to different states of the write/store/read cycle in accordance with the characteristic values taken from Fig. 2(b): 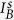 (store) = 0, 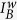 (write) = +12 mA and −12 mA, and 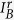 (read) = 1 mA. Then, we sequentially applied shot writing pulses of current 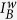 of −12 and +12 mA and measured the impedance spectra *Z*′(*f*) that are shown in Fig. 5. As one can see, there is a noticeable difference in the impedance Δ*Z*′ between *read 1* and *read 0* curves obtained at 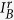 of 1 mA. The relative difference Δ*Z*′, shown in the insert, has a maximum of about 9% at the 200–300 MHz being of about 4% at all other frequencies up to the maximum measured of 2.25 GHz.

**Figure 6:**
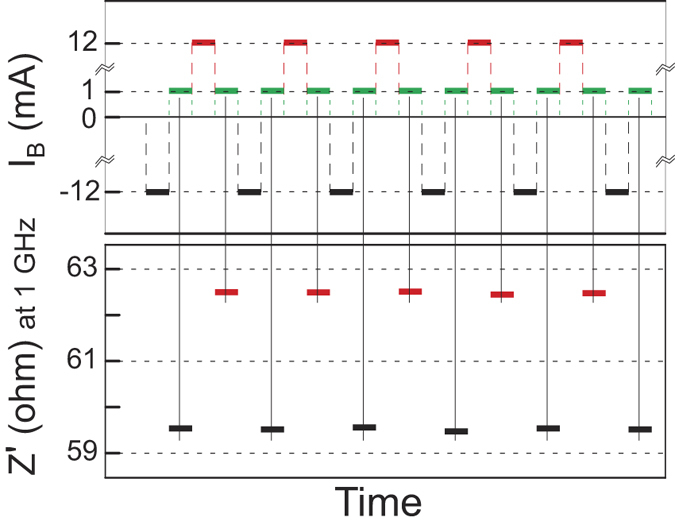
Realizations of the impedance switching cycles.

To estimate the repeatability of the impedance switching, 1000 realizations of the write and read *1/0* cycles were performed. The spread of the impedance spectra for *read 1* and *read 0* states was found to be less than 0.5% for all frequencies. A several cycles obtained at *f* = 1 GHz are shown in Fig. 6. To estimate the retention time we written the data, *logical 1* and *0*, and read out the impedance spectra (i) after 24 hours and, (ii) after 7 days and found the same spread (less than 0.5% for all frequencies) being the *High-Z* and *Low-Z* impedance curves separated as was shown in Fig. 5.

## Discussion

We have demonstrated above, both theoretically and experimentally, that the magnetization orientation in the surface layer with tilted magnetic anisotropy easy axis can be controllably switched between two stable states by applying current pulses of the appropriate sign, and can be detected by sensing the impedance. The observed hysteresis of the dependence *Z*(*I*_*B*_), usually not desirable for sensor applications, we propose to use for storing information. The principle of a such current driven impedance memory element, as has been demonstrated above, is based on (i) the static magnetization switching between two stable states by the static bias current *I*_*B*_ applied to the conductor and (ii) the dependence of high frequency impedance *Z* on the magnetization orientation as was schematically shown in Figs 1 and 2.

The proposed MI memory element has advantages compared with the currently applied multilayer magnetic memories based on magneto-resistor effect: it is much simpler – it can consist just of a single magnetic layer as schematically shown in Fig. 1. Another important advantage is the read speed. We have measured the wire impedance at frequencies up to 2 GHz. Although there exist MRAM technologies operating at higher frequencies, as for example spin-torque FMR in magnetic tunnel junctions (MTJ) which is operated at frequencies up to ten GHz^1^, the readout procedure is an asynchronous with the operating frequency and cannot be performed during its one cycle – at least tens of cycles are required to infer the magnetization state of MTJ device and retrieve it from the dc voltage response. In contrast, in the proposed MI memory one cycle of operating frequency can be enough to measure the wire impedance.

However, to compete with the existing MRAM technologies, the questions about scaling and relatively small impedance switching values are to be answered. Regarding to the scaling problem, we have demonstrated the functioning of a magnetic memory element in an amorphous microwire, as this material exhibits a very high MI effect, and the required anisotropy can be quite easily obtained. Nevertheless we believe that this concept can be extended to other materials and geometries exhibiting a high MI effect and possessing the required magnetic anisotropy. Thus, for example, a high MI effect was demonstrated in thin films and ribbons^28^,^29^. Moreover, the hysteresis behavior was shown^30^. This suggests that the proposed magnetic memory can be realized in these structures.

In regard to the relatively small measured impedance switching values, it is worth to note that the model predicts that the impedance difference between the *Read 1 s3* and *Read 0 s6* states can be considerable higher (see Fig. 2), more than 100%. Experimentally we could not achieve such values as the real switching field *H*_*sw*_ is much lower than the theoretical one because of defects^19^. Thus additional studies are required aiming to decrease the defects and, as consequence, to increase the impedance difference Δ*Z*.

Another way to increase Δ*Z* can be the application of axial magnetic field to break the symmetry of impedance dependence *Z*(*I*_*B*_). We have previously demonstrated that the application of a small external axial magnetic field *H*_*E*_ can considerably transform the impedance dependence *Z*′(*I*_*B*_) making it highly asymmetric but still hysteretic with a much higher Δ*Z*′: 100% at *H*_*E*_ = 35 A/m, 300% at *H*_*E*_ = 100 A/m^31^, and 325% at *H*_*E*_ = 145 A/m^14^. However, the impedance hysteresis region displaces from the zero current point (*I*_*B*_ = 0) that makes the memory element volatile as a continuous application of current *I*_*B*_ is required to store the data. On the other hand, for memory applications it is essential to avoid using any coil to produce axial field *H*_*E*_. Then, instead of *H*_*E*_, the core-shell (CS) biasing effect can be applied^32^. This effect manifests itself as an appearance of an effective axial magnetic field *H*_*C*_ created by remanent magnetization of the wire core that biases the outer shell. For the above shown measurements we demagnetized the core as described in the method section to set *H*_*C*_ = 0. Further, on the contrary, we magnetized the sample in axial direction in a field of 3 kA/m. It was demonstrated that this field creates an effective core remanence field *H*_*C*_ of 20 A/m in this wire^32^. Then, the surface magnetization rotation is described by Eq. 3 substituting *H*_*E*_ by *H*_*C*_ = 20 A/m.

**Figure 7:**
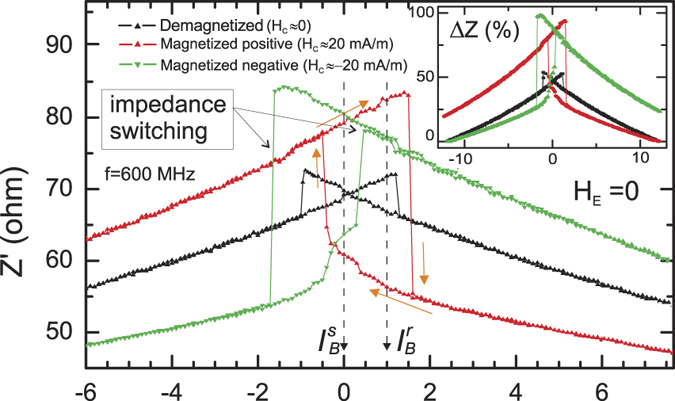
Effect of the Core-Shell biasing on the impedance dependence *Z*′ on static bias current *I*_*B*_ at *H*_*E*_ = 0. *H*_*C*_ is the effective bias field created in the other shell by remanent magnetization of the wire core. The inset shows the relative impedance change 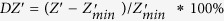 where 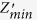 is the impedance at *I*_*B*_ = 12 mA.

**Figure 8:**
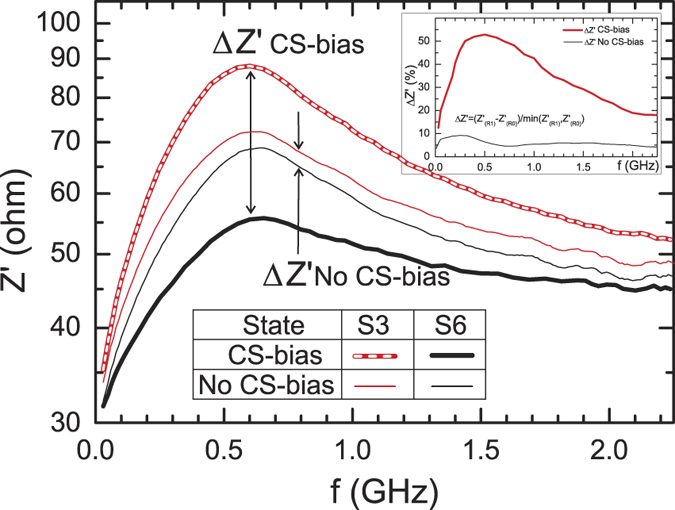
Experimental dependence *Z*′( *f*) with *I*_*B*_ = 1 mA for a magnetized with axial magnetic field of 3 kA/m. The inset shows the impedance difference Δ*Z*′ between *read 1* and *read 0* curves.

The impedance measurements *Z*′(*I*_*B*_) for demagnetized wire (also shown in Fig. 4) and for the wire magnetized with a pulse *H*_*p*_ of positive (+3 kA/m) or negative (−3 kA/m) axial magnetic field *H*_*E*_ are shown in Fig. 7. As one can see, after application of a field pulse, the impedance curve *Z*′(*I*_*B*_) transforms considerably with the impedance switching between the high (84 ohm) and the low (56 ohm) impedance states Δ*Z*′ of 50% taken at *I*_*B*_ = 1 mA and *f* = 600 MHz. The *I*_*B*_ = 0 point is still inside the hysteresis region, therefore, after removing the current, the memory element keeps its magnetization state. Moreover, as there are different impedances for *‘store 1’* and *‘store 0’* states at *I*_*B*_ = 0, the read out of information can be performed with *I*_*B*_ = 0. The impedance spectra *Z*′(*f*) measured at 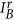 of 1 mA in the previously magnetized with *H*_*p*_ +3 kA/m wire are shown in Fig. 8. As one can see, the difference between the *high-Z* and *low-Z* impedance states is much higher (shown in the insert) in comparison with the demagnetized sample where the core-shell biasing is effectively eliminated. This axial biasing can be also performed by a permanent magnet place near the wire.

For bit detection, the schematics applied in commercially produced MI sensors can be used. The description of the sense circuits can be found elsewhere: a peak detector^33^ or synchronous *sample and hold* detection^34^ are the most common. Here the amplitude of voltage drop on the impedance element is detected and further, this voltage is compared with the reference voltage by a sense amplifier. Also, a Time-Domain Reflectometry (TDR) technique can be used to detect the wire impedance^35^.

In conclusion, we theoretically and experimentally presented a concept of a MI memory element, and demonstrated its functioning on a short piece of amorphous microwire. Here, in contrast to the usual approach where the MI effect is treated as the dependence of impedance on externally applied magnetic field *H*_*E*_, we set *H*_*E*_ = 0 and used the impedance sensitivity to the internal bias field *H*_*B*_ created by static or pulse current *I*_*B*_ flowing through the conductor. In this memory type, as in many other MRAM types, the information is stored as the magnetization orientation controlled by the applied pulse current. However, in contrast, the read out of the information is performed by sensing the high frequency impedance of the memory element. As the impedance is insensitive to the static current^19^ in a wire with circumferential anisotropy, a tilted magnetic anisotropy is required to make the impedance to be a sensitive and hysteretic function on bias current *I*_*B*_. In this case, the impedance dependence *Z*(*I*_*B*_) exhibits switchings from a high to a low impedance states when bias current exceeds the threshold value *I*_*sw*_. This hysteresis, generally undesirable for sensor applications, is proposed to use for storing information. The ascending (*store ‘1’*) and descending (*store ‘0’*) branches of the *Z*(*I*_*B*_) curves cross at *I*_*B*_ = 0, thus we applied a small static current 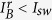 which makes the magnetization reversibly rotate in the *close-to-longitudinal* or *close-to-circumferential* directions exhibiting different impedances. The wire is able to keep the impedance value nonvolatily during a long time even when the electric power is cut off. We measured the impedance from 10 MHz up to more than 2 GHz and observed the difference of 4–9 percents between the impedance of the *store 1* and *store 0* state. The main advantages of the proposed concept are: (i) a high read cycle speed, and (ii) a simple structure of the memory element. Besides the magnetic memory, the demonstrated impedance switching and tuning can be also used in different microwave devices such as tunable filters, resonators, delayed lines, impedance matching lines etc.

## Methods

**Figure 9:**
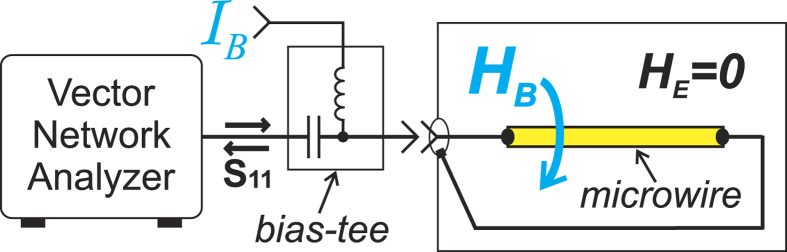
Experimental setup.

We measured the longitudinal impedance of the wire with a vector network analyzer through the reflection coefficient *S*_11_ in the frequency range 10 MHz–2.25 GHz. The impedance was measured as a function of static bias current *I*_*B*_ at an external magnetic field *H*_*E*_ equal to zero. The schematic of the experimental setup is shown in Fig. 9. Before measurements, we demagnetized the sample by performing a cycle of axial external magnetic field *H*_*E*_ oscillations with reducing amplitude to suppress the core-shell biasing^32^. In this study we investigate the dependence of wire impedance on the static bias current *I*_*B*_ (or corresponding circular static field *H*_*B*_). A maximum possible current *I*_*B*_ (field *H*_*B*_) is limited because of Joule heating of the wire. For this particular wire, the maximum current, at which the wire burns out, is 18–20 mA. The applied current of 20 mA creates a field *H*_*B*_ maximum of 310 A/m at the wire surface that is below the surface anisotropy filed *H*_*A*_ (500 A/m). Thus, the complete reversal of magnetization of the wire sample in the form *M*_*z*_(*H*_*B*_) (or *M*_*z*_(*I*_*B*_)) cannot be obtained. However, as the magnetization reversal above the switching is of rotation type and is reversible, the *Z*(*I*_*B*_) (*Z*(*H*_*B*_)) dependencies are independent on maximum field above the switching field.

## Additional Information

**How to cite this article**: Ipatov, M. *et al*. Current controlled switching of impedance in magnetic conductor with tilted anisotropy easy axis and its applications. *Sci. Rep.* **6**, 36180; doi: 10.1038/srep36180 (2016).
